# Combining Computational Modeling and Neuroimaging to Examine Multiple Category Learning Systems in the Brain

**DOI:** 10.3390/brainsci2020176

**Published:** 2012-04-23

**Authors:** Emi M. Nomura, Paul J. Reber

**Affiliations:** 1 Helen Wills Neuroscience Institute, University of California, Berkeley, CA 94720, USA; E-Mail: eminomura@berkeley.edu; 2 Department of Psychology, Northwestern University, Evanston, IL 60208, USA

**Keywords:** categorization, explicit, implicit, computational modeling, non-conscious processing

## Abstract

Considerable evidence has argued in favor of multiple neural systems supporting human category learning, one based on conscious rule inference and one based on implicit information integration. However, there have been few attempts to study potential system interactions during category learning. The PINNACLE (Parallel Interactive Neural Networks Active in Category Learning) model incorporates multiple categorization systems that compete to provide categorization judgments about visual stimuli. Incorporating competing systems requires inclusion of cognitive mechanisms associated with resolving this competition and creates a potential credit assignment problem in handling feedback. The hypothesized mechanisms make predictions about internal mental states that are not always reflected in choice behavior, but may be reflected in neural activity. Two prior functional magnetic resonance imaging (fMRI) studies of category learning were re-analyzed using PINNACLE to identify neural correlates of internal cognitive states on each trial. These analyses identified additional brain regions supporting the two types of category learning, regions particularly active when the systems are hypothesized to be in maximal competition, and found evidence of covert learning activity in the “off system” (the category learning system not currently driving behavior). These results suggest that PINNACLE provides a plausible framework for how competing multiple category learning systems are organized in the brain and shows how computational modeling approaches and fMRI can be used synergistically to gain access to cognitive processes that support complex decision-making machinery.

## 1. Introduction

An abundance of evidence has accumulated in support of the idea that there are multiple neural systems in the brain that support learning of visual categories. While category learning is often thought to rely upon conscious, goal-directed processing, there is also a significant contribution from implicit, non-conscious processes. In visual categorization, the difference in processes can be seen in comparing the conscious deduction of category membership, e.g., “that must be a cat because it has whiskers,” and the implicit learning that occurs so that after experiencing many examples of category members, a new stimulus is automatically identified as a cat. The distinction between these two processes is well-captured by the experimental categorization work contrasting rule-based (RB) and information-integration (II) category learning approaches described by Ashby, Maddox and colleagues [1,2,3,4,5]. In this work, simple experimental manipulations can lead to different categorization strategies being used by participants.

A general model describing the hypothesized neural basis of these two systems is the COVIS model (COmpetition between Verbal and Implicit Systems) model [6,7]. In this theory, the RB category learning system depends on prefrontal cortex, the head of the caudate of the basal ganglia and the medial temporal lobe (MTL) memory system while the II category learning system depends on connections between visual cortical areas and posterior regions of the caudate. The RB system depends on brain areas that support conscious, verbalizable rules for judging category membership and testing hypotheses via feedback about prior predictions. Participants who have learned a category using an RB strategy are generally able to verbally describe the rule by which they successfully categorized novel stimuli. The II system learns implicitly to partition the visual space following the category boundaries. After learning II categories, participants are generally unable to verbalize the rule that partitioned the perceptual space. Furthermore, these two systems compete to control the output response to report category membership, reflecting that learning can happen in both systems but a category judgment will depend on one system or the other.

Here a model based on the COVIS theory is described that provides candidate mechanisms for resolving competition between RB and II category learning systems, handling trial-by-trial feedback to the competing systems and attempting to capture individual participant strategy switching. The Parallel Interactive Neural Networks Active in Category Learning (PINNACLE) model is used to test theoretical predictions about category learning system interactions in the brain reflected in fMRI data and also used to guide fMRI data analysis. One of the essential challenges of this approach is that we hypothesize multiple competing systems operating in the human brain, but elements of the underlying processing are operating outside of awareness and only one response is made to provide information about the hidden processes. Functional neuroimaging provides for the possibility of revealing these hidden processes by using a computational model that makes specific predictions about the current mental state of each participant on each trial.

Prior computational modeling approaches pioneered by Maddox and Ashby have demonstrated that individual behavioral responses in categorization tasks can be used to diagnose whether participants are engaging in RB or II category learning strategies (e.g., [2]). In those modeling analyses, behavior was compared to predictions of an RB and II model separately to determine which model best accounted for the data. This allows the modeling approach to characterize the strategy of individual participants and identify participants who might initially (or persistently) use a sub-optimal strategy or choose randomly. This approach has helped to identify differences in the operating characteristics of these two systems, which has been supported by neuropsychological studies and neuroimaging studies. Rule-based category learning is sensitive to working-memory capacity and is slowed by dual-tasks that reduce available working memory [8]. While high working-memory load dual tasks do not impair II category learning, delaying feedback for as little as 2 s interferes selectively with II learning [9]. Studies of patients with memory disorders or dysfunction in the basal ganglia (e.g., Parkinson’s disease) suggest that RB-learning is impaired by damage to declarative memory systems while basal ganglia damage impairs II learning [10].

Recently, further evidence about the neural basis of the separate RB and II category learning systems has emerged from functional neuroimaging methods. Nomura *et al*. [11] contrasted neural activity associated with successful categorization of RB and II strategies and found differential activity in the medial temporal lobe (MTL) and posterior caudate for RB and II category learning respectively. The idea that these systems operate in competition with each other is supported by findings such as Poldrack *et al*. [12] in which caudate and MTL activity were inversely correlated in an implicit probabilistic category learning task. In a task that focused more on rule learning with no probabilistic component, fMRI revealed a similar pattern of opposing activity in the caudate and MTL [13]. Participants were asked to acquire a particular rule that required attending to different features of the letter stimuli. Using the feedback after each trial, they eventually discovered the rule and then applied it to subsequent stimuli. Rule learning relied upon activity in a wide network of frontostriatal areas coupled with a decline in hippocampal activity whereas in rule application, there was a rise in hippocampal activity. The authors suggest that the antagonism between the striatum and MTL may have facilitated the transition between acquisition and application of a rule. 

While the general COVIS theory includes competition between the two category learning systems, incorporating competition into a specific computational model raises several challenges about resolution of competition on each trial, strategy-switching across trials and handling feedback about response accuracy for learning within each system and to influence future competition. The PINNACLE model reflects an instantiation of potential mechanisms for these processes based on trying to identify the simplest mechanisms consistent with both behavior and fMRI data. The model is consistent with COVIS as a framework but does not attempt to account for the full range of neurobiological mechanisms potentially engaged in cortico-striatal and MTL-dependent learning.

The challenge of building an integrated model for both types of category learning is that information comes in through a single source, the visual system (controlled experimentally) and a single output is made but multiple systems operate on the information in between. The multiple systems model hypothesizes that there must be internal states in the brain corresponding to the state of both the RB and II systems that are attempting to learn the category. The system that is most consistently successful is assumed to eventually control the output behavior, but learning which system is most often correct requires an ability to evaluate and resolve competition between the two systems. In addition, participants are given feedback about the correctness of the final output, but it is not clear *a priori* how to handle a single feedback source to multiple components. For example, only the winning (currently in control) system could learn via feedback since the feedback is appropriate to the chosen response, but this raises a question of how to identify when to switch strategies. For example, participants often begin with an RB strategy for an II task and learn to shift to an II strategy over time. Before the strategy switch, no behavioral information on learning within the II system is available. A combination of computational modeling and fMRI data may provide insight into these covert cognitive processes.

The PINNACLE model of category learning provides a hypothesized structure for interactions between independent RB and II category learning systems. This model is intended to complement and extend COVIS by conjecturing additional mechanisms for handling feedback to two separate systems and also by resolving potential competition between the two systems for making responses. We test the model first by fitting individual participants’ choice behavior in RB and II category learning tasks and show that it exhibits learning and strategy switching similar to humans. The second goal of PINNACLE is to use trial-by-trial model predictions to test hypotheses about neural system engagement derived from fMRI data. By identifying specific trials of interest (e.g., where competition between the two systems should be maximal according to the individual participant model fit), additional brain regions were identified as likely sources for the neural mechanisms needed to instantiate the two parallel models of category learning in the brain. In addition to providing hypotheses about the organization of multiple categorization systems within the human brain, this approach thus shows how computational modeling and functional neuroimaging data can be integrated to harness the inferential power of each approach together.

## 2. Model Description

**Figure 1:**
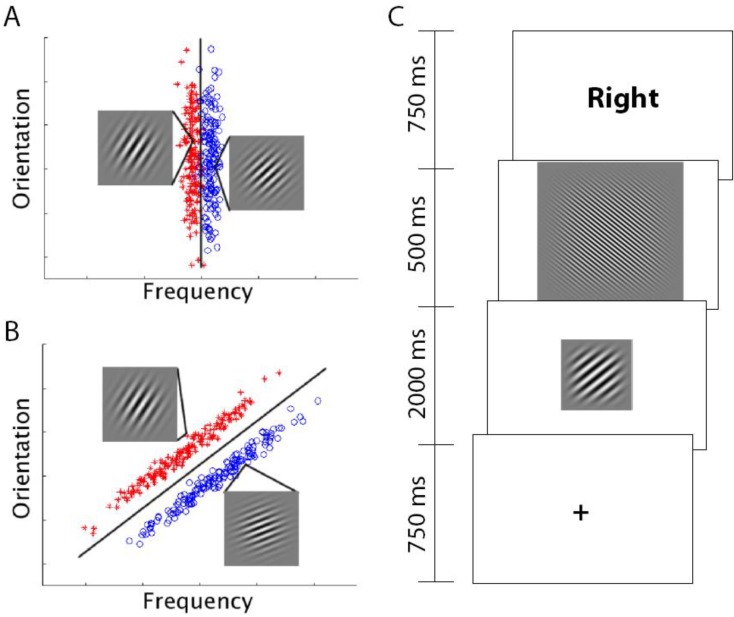
RB (**A**) and II stimuli (**B**). Each point represents a distinct Gabor patch (sine-wave) stimulus defined by orientation (tilt) and frequency (thickness of lines). In both stimulus sets, there are two categories (red and blue points). RB categories are defined by a vertical boundary (only frequency is relevant for categorization) whereas II categories are defined by a diagonal boundary (both orientation and frequency are relevant). In both RB and II stimuli there are examples of a stimulus from each category. (**C**) Schematic of a single trial. A fixation point is followed by the to-be-categorized-stimulus (either RB or II depending on the subject), then a short visual mask that is followed by the feedback. The subject responded “category A” or “category B” during the 2 s the stimulus was on the screen using hand-held buttons. The length of the inter-trial interval (ITI) was pseudorandom and based on between zero and five 4-s “fixation-only” trial periods arranged to maximize the separability of the measured hemodynamic response to stimulus trials.

For visual categories, category structure can be efficiently described as partitioning perceptual space and assigning category labels (or motor responses) to regions that encompass a collection of similar stimuli. A mathematical description of this approach is the core of the decision-bound theory (DBT) of category learning first described by Ashby and Gott [14]. Under DBT, when presented with a to-be-categorized stimulus, participants determine in what region the stimulus has fallen and produce the associated response. Feedback about the accuracy of the judgment is used to adjust the boundaries to improve future categorization performance. In this approach, learning the categories is equivalent to identifying the optimal decision-boundary that separates the categories in the perceptual space. In most studies of RB and II category learning, stimuli are constructed in a 2-dimensional space with continuous dimensions. An example of 2-dimensional stimulus space and the corresponding category bounds is shown in Figure 1. 

Both RB and II category learning can be described by DBT models. A key difference is that for RB categories, the decision boundary is typically along a single dimension that allows for a simple verbal description of the category rule. A vertical line partitioning the stimulus space is equivalent to a verbal rule referencing that value on the key dimension as “less than this value is an A, greater than this value is a B.” Tasks that lead to II category learning generally have decision boundaries that require integration of information across the dimensions. A diagonal line through the stimulus space provides partitioning into two categories, but there is no simple verbal description of the stimuli that characterizes this boundary.

**Figure 2:**
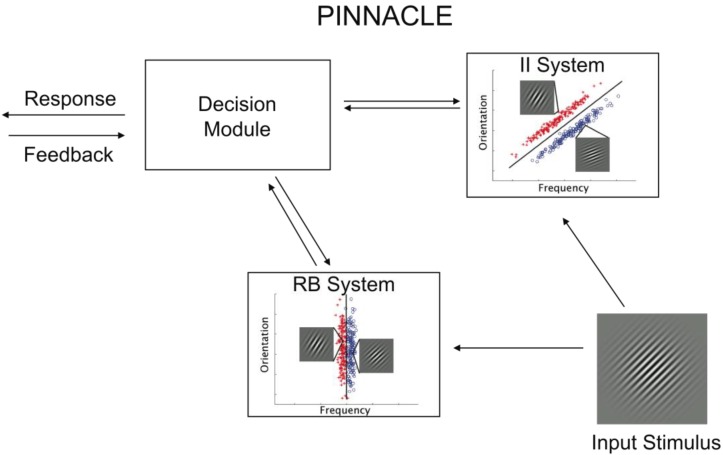
Schematic of the PINNACLE computational model and the accompanying free parameters. Stimulus information is fed into the RB and II systems and evaluated separately. A categorical decision is made within each system, but the decision node adjudicates between the systems according to their relative confidence levels. After a response is made, feedback returns to the system that made the response, and in the case of negative feedback, the representation is updated.

Prior modeling work has shown that DBT descriptions are effective at capturing the process of visual category learning [1,2,14]. However, this approach does not provide a framework for competition between RB and II models. The PINNACLE model is based on two separate DBT models, one for representation of RB learning and one for II learning. These two representations are further organized into a system for resolving competition, strategy switching and appropriate handling of feedback (see Figure 2). While the organization of these systems in PINNACLE is adapted from COVIS, the resolution of competition (and strategy switching) and the methods for handling trial-by-trial feedback are specific to the implementation of PINNACLE and derived from our behavioral data (Section 3). The representation of both RB and II component category learning systems as simple DBT models is meant to reflect the general operation of these components but not the internal processes of MTL-PFC interactions (RB) or cortico-striatal (II) circuits.

### 2.1. DBT Details

For the purpose of the PINNACLE model, stimuli are represented as points in 2-dimensional space reflecting the orientation and frequency of the sine wave gratings. The category representations are maintained as the coordinates of a single line that bisects the perceptual space into two regions reflecting the two categories (A, B). In addition, a shaping parameter (PS) is used that captures perceptual noise and confidence in category label judgments following previous DBT models of category learning (e.g., [15]) that have incorporated a similar measure referred to as “perceptual noise”. For a given input stimulus, the probability of being in category A is calculated by the distance of the stimulus coordinates to the boundary line (Equation 1). Here, the RB_bound_ is a vertical line partitioning the space based on the stimulus frequency and the II_int_ is the intercept defining a diagonal line with slope = 1.0 partitioning the category space on both dimensions. In both calculations, the *x_i_* and *y_i_* variables are the current *x*- and *y*-coordinates of a stimulus. 

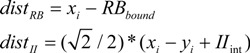
(1)


The shaping parameter determines the spread of a 3D Gaussian applied to this point and bisected by the boundary line. The percent of this distribution that falls in the A section of the perceptual space is the probability the stimulus is a member of category A (Equation 2). 

For positive values of *z* where *dist* is derived from Equation 1,

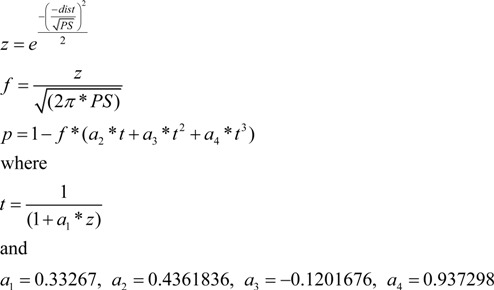
(2)


The constants (*a*_1–4_) in this equation were defined in Ashby’s original chapter on probability distributions [16]. This probability, *p*(A), increases as the stimulus is further from the boundary (meaning it is more clearly in the A category). For larger values of PS, the spread of the Gaussian distribution is wider meaning that more of the distribution crosses the boundary and the estimate of *p*(A) is reduced, essentially reflecting lower confidence. For lower values of PS, more of the distribution falls within a single category region and estimates of *p*(A) are increased, reflecting greater confidence in the judgment. 

For the RB representation in DBT, the category boundary is restricted to being a single vertical line (Equation 1). This simplification allows for representation of the RB stimulus conditions used in our experimental tasks. A full representation of RB category learning would include processes associated with considering and testing a much broader range of hypotheses as well as explicit memory for prior stimuli and feedback. The minimalist RB representation used in PINNACLE approximates this for a narrow range of hypotheses (ones about the category being based on the frequency of the grating) and serves to distinguish judgments made about these kinds of hypotheses from II categorization.

The II representation is a nearly identical, separate DBT representation in which the category boundary is restricted to be a single diagonal line of slope 1.0 (the intercept can vary; Equation 1). Like with RB, a minimalist representation is chosen that matches the experimental stimuli and also matches the number of free parameters used to represent the range of possible RB categories. A fuller representation of II category learning should provide a mechanism for arbitrarily labeling regions of perceptual spaces with more dimensions [17]. The simple and limited II representation used here should again approximate the narrow range of hypotheses that would account for II category learning and serve to distinguish it from RB categorization. A more comprehensive extension of PINNACLE would incorporate a flexible model of hypothesis testing as part of the RB module and hypothesize a neural mechanism for learning complex and nonlinear II categories through feedback.

### 2.2. Learning Details

Since PINNACLE is fundamentally a model of the learning process, a key component of the model is how the parameters of component DBT descriptions change on a trial-by-trial basis. When a stimulus is encountered, the current state of the DBT parameters indicates the probability that the stimulus is in category A, *p*(A) (Equation 2). When feedback is provided that indicates the category membership of the stimulus, the model parameters are adjusted accordingly (Equation 3).

*RB_update_* = *RB_lr_* * │ *RB_current_ − RB_bound_* │ *II_update_* = *II_lr_* * │ *II_current_ − II_bound_* │ PS_update_ = PS * PS_lr_ (3)

If the stimulus was in the predicted category, *i.e*., A if *p*(A) > 0.50; B if *p*(A) < 0.50, the decision boundary is assumed to be correct and PS is reduced, leading to greater confidence of the category prediction should any similar stimuli be encountered subsequently. If the stimulus was in the alternate category, *i.e*., the prediction was incorrect, the decision boundary is moved incrementally (RB_update_, II_update_) in the direction that minimizes the error and PS is increased (PS_update_), leading to lower confidence in predictions about future stimuli (note that in Equation 3, the direction described as calculated by reference to the optimal boundary, which is not available to the model but is instantiated as moving away from the current stimulus to produce an identical error-reduction effect).

For each of the two DBT models (RB, II) we allowed for independent learning rate parameters (RBlr, IIlr) for each system for the size of the increment for adjusting the category boundary. Although separate shaping parameter values were maintained for each system, only one learning rate parameter (PSlr) was needed to fit to behavioral data.

### 2.3. System Interaction

One of the key elements of the PINNACLE model is providing a framework for modeling the operation of two simultaneous category learning systems. Since only a single output response can be made, it is necessary to hypothesize a mechanism for handling the multiple sources of information; we refer to this mechanism as the Decision Module. This is implemented as a simple model that favors the more confident system proportional to the ratio of the odds (Equation 4) implied by the confidence of the system plus a noise parameter (Equation 5).

*Odds* = *conf* / (1.0 − *conf*) (4)

where *conf* is equal to the maximum RB or II system probability (of A or B).

To model the decision noise associated with the system selection, a random number (ε) is selected from a Gaussian distribution defined by its standard deviation (DMnoise). The system with the higher odds value after the addition of the random number (ε) is selected to make the category decision (Equation 5).

max(Odds_RB_ + ε, Odds_II_) (5)

Both the RB and II systems provide independent predictions on each trial which can differ in category and confidence. For example, the RB system may judge a stimulus as *p*(A) = 0.8 and the II system predict *p*(A) = 0.7. In this case, the RB system is more confident and more likely to drive the output response (the fact that the II system prediction is consistent has no effect). As another example, the RB system may predict *p*(A) = 0.8 and the II system predicts *p*(A) = 0.1. In this case, the II system is making a more confident prediction that the correct category is B. The noise added to the decision process allowing for occasionally selecting the less confident system (proportional to the size of this parameter). 

Typically, one of the models is making more confident predictions and tends to be consistently selected, leading to the expression of either an RB or II strategy across trials. The noise parameter (DMnoise) allows for occasional sampling of the other approach, which facilitates strategy switching. It is fairly common for participants to start with one strategy, e.g., RB, and after some trials of feedback, switch to the other strategy (II, in this case). The primary mechanism for this in the model is that when feedback indicates that a response was incorrect, the shaping parameter is increased, leading to a general loss of confidence for that model. This lets the other, “off system” model compete more effectively on subsequent trials and potentially leads to a strategy change.

### 2.4. Feedback

The Decision Module provides a mechanism for resolving competition that will allow for strategy switching based on correct/incorrect feedback on each trial. However, this system raises an important question of how feedback should be handled in a multi-system model. On each trial, both systems make a prediction, one wins the competition, a response is selected and made and experimental feedback is provided for that trial. There is a potential credit assignment problem left open by this process, particularly with respect to the “off system” (*i.e*., the system that provided the prediction not used to drive the response). One possibility is that only the active system that drove the response is adjusted by feedback. However, if the off system is making consistently accurate predictions, it would be more efficient to use the feedback to improve its predictions as well because this would lead to more rapid and accurate strategy switching. In addition, it is possible that positive and negative feedback might be handled differently in the two types of learning. In hypothesis testing (RB), disconfirming evidence is particularly important for changing hypotheses. In contrast, II learning is thought to depend on dopamine-gated plasticity in cortico-striatal circuits that might be more sensitive to positive feedback (which should produce transiently elevated dopamine levels). Rather than embed an assumption about the mechanism for handling feedback, attempts to answer these questions were addressed via comparative simulations with a number of different feedback systems in PINNACLE to identify which feedback-handling model provided the best account for the behavioral data (Section 3).

The modeling results are separated into two sections. An initial set of simulations in Section 3 show that PINNACLE fits human behavior over two experimental datasets and variants of the model, reflecting different assumptions were competitively evaluated to identify the best fitting alternatives within the general model architecture. The winning version of PINNACLE was then used to model the cognitive state of individual participants on a trial-by-trial basis from two fMRI experiments in Section 4. This modeling was used both to enhance the specificity of the fMRI data analysis and also to identify neural correlates for hypothesized internal components of the PINNACLE model.

## 3. Testing PINNACLE with Experimental Data

For the first set of simulations, PINNACLE is fit to groups of participants from two prior published category learning experiments [11,18]. Both experiments were studies of the neural correlates of category learning in which participants learned either RB or II category structures while fMRI data were collected. For these initial simulations, only the behavioral data were used to test variations in the structure of the PINNACLE model and comparisons were made between groups of participants and groups of PINNACLE simulated runs. In Section 4, PINNACLE was used to predict the cognitive state of each participant on each trial in order to enhance the analysis of the fMRI data.

The first contrast here compared PINNACLE as described above with a simplified system that only includes a single learning parameter for both RB and II category learning. This simplified model is one possible way to construct a single-system model of category learning, *i.e*., that categories of both boundary-type can be represented, but the learning is essentially identical for both. This simplified model does not account for human data as well as the multi-system model of PINNACLE does, providing some additional evidence that the multiple system structure of PINNACLE is necessary to account for behavior. The second simulation compared different potential feedback mechanisms for fit to human behavior to identify if feedback should be returned to both systems on every trial (regardless of competition) and if positive/negative feedback should be handled differently.

### 3.1. Participants

In Dataset 1, 34 healthy, native English-speaking, right-handed adults (15 males, 19 females) and in Dataset 2, 33 healthy, native English-speaking, right-handed adults (10 males, 23 females) were recruited from the Northwestern University community for participation. Reports of general category learning performance from both datasets have been published previously [11,18]. All participants gave informed consent according to procedures approved by the Northwestern University Institutional Review Board and were compensated for their time. Participants were randomly assigned to either the RB or II group in both cases.

### 3.2. Materials

In both datasets, stimuli were circular sine wave gratings (Gabor patches; see Figure 1) that varied in spatial frequency (thickness of lines) and orientation (tilt of lines) as in Maddox *et al*. [19]. Participants were instructed to place each stimulus into one of two categories and to try to learn these categories over time based on the feedback given after each trial. The only difference between the RB and II groups was in the boundary that defined the categories. The stimulus space for both the RB and II groups can be thought of in two dimensions, spatial frequency on the *x*-axis and orientation on the *y*-axis. For the RB group, the stimuli were divided into categories based on a vertical decision boundary such that category membership depended only on the spatial frequency of the sine wave grating (Figure 1A). For the II group, the categories were defined by a diagonal decision boundary that required integration of spatial frequency and orientation information (Figure 1B). The stimuli used for the two datasets differed in the variance across the category structures with within-category variance being higher in Dataset 2 providing a set of stimuli sampled over a wider range of the whole perceptual space.

### 3.3. Procedure

On each trial, a fixation cross was presented for 750 ms followed by a single stimulus that was presented for 2 s and during this time, participants indicated to which category they judged the stimulus belonged. Stimulus offset was followed by a 500 ms visual mask and feedback for the participant’s choice (“Right”, “Wrong”) was shown for 750 ms. Participants were warned (“Time”) if they had not made a response during the 2 s the stimulus was on the screen. A total of 320 categorization trials were performed by each participant divided amongst 4 80-trial blocks. An equal number of fixation-only trials were pseudo-randomly interspersed between stimulus trials to maximize the separability of the measured hemodynamic response.

### 3.4. Model Simulation

To simulate the performance of participants in the RB and II experimental groups, PINNACLE received stimulus information in the same trial order as the human subjects. PINNACLE predicted category membership, received feedback and updated the representation of the category knowledge on each trial. At the beginning of the simulated experiment, like a naïve participant, the model has no advance knowledge of the underlying category structure condition to which it has been assigned. Non-random starting positions for both the RB and II systems were used according to the average dimensions of the first two distinct subject responses. The perceptual shaping parameter was initially set at a relatively high value representing the lack of confidence in the initial category boundary.

In addition, to simulate some of the variability in human performance, after selecting the category system to use, the actual category choice reflected the predicted *p*(A) for that system. Thus, if the more confident system indicated *p*(A) = 0.8, 80% of the time A was chosen for response and 20% B was chosen (probability matching, [20]).

For each run, 100,000 PINNACLE simulations were run with a specific set of initial free parameter values. The average accuracy and standard deviation across the group of simulations on each block of the RB and II conditions was compared to the human behavior observed in the experiment. Matching the data was evaluated as producing the lowest mean squared error across the 8 group mean performance points (4 blocks for both the RB and II conditions) and the 8 standard deviation points (although the standard deviations were weighted as 0.001 as important as means to emphasize fitting overall performance rates).

A downhill simplex search algorithm [21] was used to identify the best possible set of free parameters for each form of PINNACLE compared. The free parameters fit this way included: (1,2) learning rates for the category boundary for each of the RB and II systems; (3) learning rate of the perceptual shaping parameter; (4) the magnitude of the decision choice noise parameter; and (5) the initial value of the perceptual shaping parameter. Only one set of free parameter values was identified and was fit for both groups of participants (RB and II conditions) across both experiments, even though different category stimulus distributions were used.

### 3.5. Simulation 1

An initial simulation contrasted PINNACLE with the best possible fit of a simplified model that only used a single learning rate parameter for both RB and II category learning. Although PINNACLE has relatively few free parameters to fit, this first comparison evaluates whether a 4-free-parameter model can generally fit human group mean performance data.

**Figure 3:**
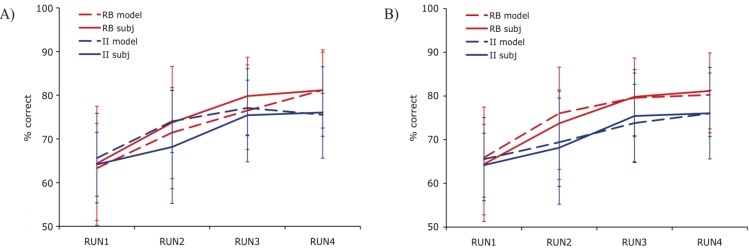
Comparison of PINNACLE model fits with a single or separate learning parameters. (**A**) Model accuracy *versus* RB and II participant group accuracy for the best fitting single learning parameter model. The model learns the basic visual category learning task, but cannot capture the differences in human RB and II category learning; (**B**) Model accuracy *versus* RB and II participant group accuracy with two learning parameters. This model accurately fits both the learning rate and between-subject variability in category learning for both RB and II systems.

Both PINNACLE and the simplified 4-parameter model were fit to the behavioral data using the downhill simplex search to maximize the fit. The best fitting parameter values produce group mean performance shown in Figure 3. The 5-free-parameter version of PINNACLE can be seen to much more accurately characterize both RB and II category learning curves across the two experiments. This suggests that a simplification of PINNACLE is unlikely to provide an account of human RB/II category learning and suggests that accurate human category learning modeling requires separate learning rates for the two types of learning. This minimal fitting parameter set does not include parameters such as an initial bias towards RB learning to capture that observed tendency [6]. That phenomenon may be embedded in the relatively higher learning rate for the RB system obtained by the fitting process (Table 1) suggesting that this bias may not require a specific additional parameter.

**Table 1:** Comparison of feedback models (A) 4 parameter model fits (B) 5 parameter model fits. In the following tables, the data is sorted according to fit value (sum of squares), with increasing values indicating worse fits to the data. The version of the feedback model corresponds to the model number in Table 2. The result of the minimization procedure was a set of parameters: RBlr (learning rate of the RB system), IIlr (learning rate of the II system), PSlr (learning rate of the perceptual shaping parameter), PS (starting perceptual shaping parameter) and DMnoise (standard deviation of the Gaussian noise distribution parameter).

Feedback	Fit value	RBlr	IIlr	PSlr	PS	DMnoise
2	14.6242	0.1703	0.0288	0.5382	145932	1.2043
1	29.0467	0.5203	0.0357	0.3689	99857	7.1001
6	44.4372	0.0601	0.3661	0.5796	156153	15.5632
3	50.3389	0.9612	0.3996	0.3906	172913	3.4329
5	84.9332	0.5259	0.0169	0.2025	176041	7.2375
4	160.0943	0.8720	0.1061	0.1186	64529	0.2630
12	178.4442	0.7514	0.1754	0.2840	56990	6.6114
8	213.5469	0.0408	0.1129	0.3195	119288	31.2168
11	263.6949	0.9084	1.0447	0.3700	210546	18.4908
9	305.5924	0.7797	1.3193	0.4274	201687	43.6479
7	809.5260	1.5725	0.0318	0.5911	163536	20.0740
10	1102.3731	0.9352	0.4984	0.4274	209661	38.9850

**Table 2:** Different feedback mechanisms for the PINNACLE model.

Model Number	Description
1	If the RB system is selected, the RB system gets feedback. If the II system is selected, the II system gets the feedback. This is true on both correct and incorrect trials.
2	Both systems get feedback on every trial.
3	Feedback only occurs on correct trials and goes to both systems.
4	Both systems get feedback on every incorrect trial.
5	If the RB system is selected, both systems get feedback. If the II system is selected, only the II system gets feedback.
6	If the II system is selected, both systems get feedback. If the RB system is selected, only the RB system gets feedback.
7	If the RB system is selected on an incorrect trial, both systems get feedback. Only the II system gets feedback on every other trial.
8	If the II system is selected on an incorrect trial, both systems get feedback. Only the RB system gets feedback on every other trial.
9	If the RB system is selected on a correct trial, it receives feedback. If the II system is selected on an incorrect trial it receives feedback.
10	If the II system is selected on a correct trial, it receives feedback. If the RB system is selected on an incorrect trial, it receives feedback.
11	Feedback only occurs on correct trials and goes to the system that was assigned to the response.
12	Feedback only occurs on incorrect trials and goes to the system that was assigned to the response.

### 3.6. Simulation 2

As noted above, the structure of PINNACLE does not imply a strong hypothesis about which possible feedback system should be incorporated into the model. In PINNACLE there are a number of different ways that the feedback can affect the internal state of the model. A key distinction among methods of processing feedback is whether feedback information is available to both systems. Although the RB and II category learning systems make independent predictions about the category membership of the presented stimulus, only one response can be chosen by the Decision Module. Feedback is based on this response, creating a credit assignment problem, particularly if a strategy switch will eventually be needed. A simple approach is to assume that only the system driving the response is updated as a result of feedback. That is, both RB and II predict category membership, but if the RB system’s response is chosen in the Decision Module, only the RB category representation is updated based on feedback to the response. A major alternate approach is that feedback is available to both systems. In this second approach, the “off-system” (that did not drive the response) learns covertly, improving predictions based on feedback even when it did not directly influence the prediction. The best example of this sort of covert learning is when the RB and II systems independently predict the same categorical response and can then utilize the feedback similarly (*i.e*., RB and II predict “A”, receive feedback that the response was correct). We explore the question of off-system learning empirically in Section 4 with fMRI data.

In addition, there is some behavioral evidence that feedback may be handled differently by the RB and II systems. For RB learning, a working-memory load impairs the use of feedback, suggesting the involvement of prefrontal executive control processes. For II learning, a delay between the stimulus and feedback impairs learning, possibly due to the time course of dopamine release to drive learning [22,23]. We therefore also considered the possibilities that only either the RB or II system could selectively use feedback in the “off-system” state separately. The importance for dopamine in II learning (due to its dependence on cortico-striatal circuits) also raises the possibility that positive and negative feedback might be handled differently across systems. The role of dopamine in positive feedback might mean it is more critical to II learning, whereas the hypothesis testing critical for RB learning might be more influenced by negative (disconfirming) feedback. These hypotheses are further motivated by the observation that II learning proceeds more quickly when participants are given positive feedback [24] and RB learning proceeds more quickly with both positive and negative feedback [25].

Twelve different versions of the feedback handling mechanism were instantiated following the different possibilities of positive and negative feedback and off-system learning (or not) (Table 2). Each model was fit optimally to the behavioral data using the same downhill simplex algorithm. Total optimal fit to all the participants in both groups was evaluated across all 12 models to identify which system best accounted for the behavioral performance. The best fitting model was the mechanism that allowed for learning in both systems on all trials regardless of the valence of feedback. In this case, the category representations in both systems receive feedback and update accordingly, even if that means reinforcing an incorrect category bound. The second best model was the model that assumed competition affected feedback such that no learning occurred in the “off-system”. The sum-of-squares fit value for the best model was 50% better than the second best model (and all other models were considerably worse) (Table 1).

The better fit of the PINNACLE model that allows learning in the system that did not drive the response suggests a specific hypothesis about neural activity associated with category learning. Although this learning process is essentially entirely covert, because it does not directly influence the outcome of the current trial, this hypothesis could potentially be tested with fMRI to examine the neural correlates of activity during category learning. In the next section, we describe how PINNACLE was used to identify critical trials to sort an event-related fMRI design to evaluate this hypothesis.

## 4. Using PINNACLE Model Fitting to Enhance fMRI Data Analysis

The first simulations in Section 3 were aimed at identifying the number of free parameters and feedback structure that provided the best fit between groups of PINNACLE simulations and groups of human participants. For a second set of simulations aimed at incorporating fMRI data, PINNACLE was fit to individual participant’s behavior on a trial by trial basis. In this case, PINNACLE provides a hypothesis on each trial of the internal cognitive state of the participants with respect to the RB, II and competition resolution systems. This state information can be used to provide novel methods of post-hoc trial sorting to test additional hypotheses about the operation of competing category learning systems in the human brain.

### 4.1. fMRI Acquisition and Analysis

For both experiments, fMRI data were collected using a Siemens TRIO 3.0 T MRI scanner equipped with a transit/receive head coil while participants performed the categorization task. Whole-brain, gradient-recalled EPI (35 axial 3 mm slices, 0 gap) were collected every 2 s (TR = 2000; TE = 25 ms; flip angle = 78°; 22 cm FOV; 64 × 64 acquisition matrix; resulting voxel size = 3.44 × 3.44 × 3 mm) for 330 volumes in each of four scans. For anatomical localization, high-resolution, 3D MP-RAGE T1-weighted scans (voxel size = 0.859 mm × 0.859 × 1 mm; 160 axial slices) were collected for each participant following the functional runs.

The functional images were first co-registered through time to correct for motion using a 3D alignment algorithm [26]. Voxels with low signal (<100 units, 30% of mean signal) or excessive sudden signal change were eliminated (>30% in 2 s) and the EPI data were smoothed (6.9 mm FWHM Gaussian kernel). Data were transformed to standard stereotactic space (MNI 305; [27]). Estimates of trial-locked evoked activity were made for the period of 4–12 s after stimulus onset to account for hemodynamic delay with overlapping responses deconvolved via a general linear model. 

In addition to the whole-brain analysis, the ability to identify anatomical boundaries for two critical regions hypothesized *a priori* to be important for category memory enabled a specific region of interest (ROI) analysis in the hippocampus and the caudate. For each participant, ROIs were drawn following anatomical boundaries that are visible on structural MRI. The MTL ROIs were drawn using boundaries that are described elsewhere [28,29]. The caudate ROIs were drawn according to known neuroanatomical boundaries separating the caudate from the surrounding white matter and ventricles. Each individual’s ROIs were then aligned using the ROI alignment (ROI-AL) method described in Stark and Okada [30]. This method optimizes regional alignment at the expense of whole-brain alignment allowing for more precise localization and enhanced statistical power. Of particular interest was to test whether these two regions play different roles in RB and II categorization, *i.e*., whether there was significantly different activity associated with successful categorization in the RB and II groups. Separate reliability thresholds for contrasts between the participant groups within the ROIs were identified by additional Monte Carlo simulations (the MTL ROI volume was 21,500 mm^3^, the caudate ROI was 11,000 mm^3^; note that this method matches the shape as well as providing a “small volume” correction for the ROI volumes). Within the targeted ROIs, an alpha level of 0.05 is met by requiring clusters for which each voxel exhibited *t*(24) > 2.0 to be at least 700 mm^3^ in volume for the MTL, 600 mm^3^ for the caudate.

### 4.2. PINNACLE Fit

For each participant, a set of free parameters for PINNACLE was identified that provided the best fit to participants’ individual behavioral data using maximum likelihood estimation. Starting values for the DBT models (category boundaries) were initialized to the average position of the first two stimuli encountered. The other PINNACLE parameters were set using the downhill simplex method. On each trial, both the RB and II systems of PINNACLE make predictions about category membership. The system that most closely matched the participant’s choice data was assumed to have been responsible for the category judgment on that trial. The closer that prediction was to 1.0 for the chosen outcome, the better the model prediction on that trial was judged to be. Feedback based on that response was used to update the category representations for the next stimuli just described above. In this approach, the likelihood of the data given the model is the product of the probabilities of the category choices on every individual trial. The downhill simplex search algorithm was used to identify the set of free parameters that made the data maximally likely for PINNACLE. Note that this effectively sets the free parameters to maximize the fit of the model across all 320 trials of performance essentially simultaneously.

Once the best individual parameters were identified, the model was run with these parameters to provide an estimate of the state of the DBT models and Decision Module on each trial for that individual participant. These were used to analyze the fMRI data by creating post-hoc sets of trials based on conditions where trials clearly reflect the expression of selectively RB or II category use, where there should be high levels of competition between systems and to explore neural activity associated with the system not driving the output category judgment response. In each of these cases, the analysis is depending on the model to characterize trials that cannot be easily distinguished simply on the basis of choice behavior. This type of analysis is the primary goal and benefit of combining fMRI and computational modeling approaches.

### 4.3. Result 1: Best Fitting RB and II Trial Activity

**Figure 4:**
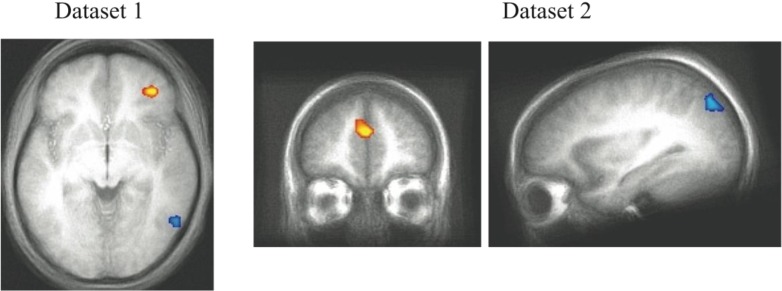
Correct trial activity for the best fitting RB *versus* best fitting II blocks. In Dataset 1 and Dataset 2, a region in right PFC is more active for best fitting RB blocks (red) and a region in right posterior visual association cortex is more active for best fitting II blocks (blue).

The fit value for a given block of 80 trials amounts to the sum of the individual trial fits in that block. To identify the best examples of RB and II trial activity irrespective of the group the subject was assigned, we restricted the analysis to the best fitting blocks of data defined as the top third of all blocks. Within these blocks, a contrast of correct RB and II trials identified a number of regions of activity similar to those found with DBT-based modeling [18]. That is, trials in which PINNACLE predicted the RB system was successfully engaged in learning were compared to trials where the II system was successfully learning (Figure 4). A region in the right PFC (Dataset 1) and medial PFC (Dataset 2) was more active during correct RB than II trials. The opposite effect was observed in right posterior visual association cortex where activity was greater during correct II than RB trials (Datasets 1 and 2). The contrast of activity associated with correct RB and II trials should emphasize brain regions associated with effective expression of those strategies. In our previous work, the contrast between successful and unsuccessful trials found differential activity in the MTL and posterior caudate, which was not observed in the current success-only contrast (in best-fitting blocks, there are too few unsuccessful trials to examine this contrast). This may reflect covert activity in the “off system” (e.g., II system activity during RB trials) that weakens this difference and will be examined more directly below.

These areas of system-specific cortical activity are consistent with prior hypotheses about the neural basis of RB and II category learning. The RB system is thought to rely upon working memory and executive attention, functions that are known to engage the PFC and anterior cingulate cortex. These regions are also highly interconnected with the head of the caudate, which is hypothesized to play a role in RB category learning [10]. Learning in the II system is thought to depend on reciprocal loops between striatal and posterior cortical visual regions in which feedback processing the basal ganglia influence changes in sensory cortical processing (a ventral temporal region in Dataset 1, superior occipital cortex in Dataset 2). In our previous analyses [11,18], the role of the basal ganglia in the learning process was observed by contrasting trials on which the II category judgment was successful. In the analysis reported here, the best fit blocks tend to have very few unsuccessful trials making that contrast ineffective. The question of whether basal ganglia activity differs during RB and II conditions depends on whether the off system is covertly attempting to learn the category as well which will be considered below (Section 4.5). The cortical regions found here that are thought to contribute to the RB and II networks were not identified in previous analyses that focused on group conditions (assignment to RB or II category structures) and success-based contrasts between trials [11]. However, the fact that participants do not always consistently express the strategy ideal to their condition (e.g., participants strategy switch between RB and II at various points regardless of condition) makes contrasts based on experimental group assignment vulnerable to loss of sensitivity due to averaging strategy use. Using PINNACLE to more precisely assess the trial-by-trial state of the RB and II systems to provide more accurate trial sorting appears to lead to increased sensitivity to detecting the neural components of the two category learning systems.

### 4.4. Result 2: Decision Module Activity

An important element of the architecture of PINNACLE is the Decision Module that is necessary to adjudicate competition between the RB and II systems. We hypothesize that each category learning system provides information about the membership of a stimulus to be categorized to a brain region in which the competition between systems is resolved and a motor response is then selected. This brain region will be active on virtually every experimental trial and therefore tend not to be identified in contrasts based on the categorization strategy or success. However, trials in which there is more competition between systems might lead to greater activity in this region due to more neural activity being required to resolve which information to rely on to make the response.

In the following analysis, PINNACLE was used to identify trials where there was greater competition between the RB and II systems. The most difficult decision between the RB and II systems should occur when both systems are highly confident and make different predictions about the membership of the stimulus. We defined high-competition (C) trials as ones in which both RB and II systems are >75% confident of category membership with different predictions. Trials in which one system is highly confident (>75%) and the other is not typically reflect conditions in which one system has come to dominate the strategy and is likely the correct one for the category structure administered experimentally. These non-competitive (NC) trials should be very easily resolved within the Decision Module and are contrasted with the C trials. Trials in which both systems were confident and consistent, or trials where neither system was confident, were left undefined and not included in this analysis. These undefined trials typically occur early in training when both systems have reasonable models of the category boundary and disconfirming evidence for the incorrect system have not been encountered. Across both datasets, C trials varied from 23% to 26% while NC trials occurred on 41%–42% of all trials. Accuracy and reaction-time, however, was generally similar for both trial types across blocks with the exception of accuracy in Dataset 2 (values in Table 3). In Dataset 1, accuracy for C and NC trials did not differ across blocks (Block 1: *t*(24) = 0.58, *p* = 0.59; Block 2: *t*(24) = 1.14, *p* = 0.28; Block 3: *t*(24) = 1.43, *p* = 0.175; Block 4: *t*(24) = 1.03, *p* = 0.32). In Dataset 2 accuracy for C and NC trials did not differ in 2 out of 4 blocks (Block 1: *t*(24) = 1.23, *p* = 0.24; Block 2: *t*(24) = 2.26, *p* = 0.04; Block 3: *t*(24) = 3.1, *p* = 0.01; Block 4: *t*(24) = 0.9, *p* = 0.38). In Dataset 1, reaction-time for C and NC trials did not differ across blocks (Block 1: *t*(24) = 0.87, *p* = 0.43; Block 2: *t*(24) = 1.4, *p* = 0.2; Block 3: *t*(24) = 0.62, *p* = 0.54; Block 4: *t*(24) = 0.85, *p* = 0.41). Likewise, in Dataset 2, reaction-time for C and NC trials also did not differ across blocks (Block 1: *t*(24) = 0.68, *p* = 0.52; Block 2: *t*(24) = 0.32, *p* = 0.76; Block 3: *t*(24) = 0.19, *p* = 0.85; Block 4: *t*(24) = 1.41, *p* = 0.17).

**Table 3:** Average accuracy and reaction-time for competition (C) and non-competition (NC) trials in Dataset 1 and Dataset 2.

			Block 1	Block 2	Block 3	Block 4
Accuracy	Dataset 1	C	74%	91%	90%	91%
		NC	69%	85%	92%	85%
	Dataset 2	C	80%	83%	87%	80%
		NC	72%	73%	79%	72%
RT	Dataset 1	C	1.26 s	0.96 s	0.85 s	0.88 s
		NC	1.23 s	1.01 s	0.84 s	0.86 s
	Dataset 2	C	1.17 s	1.13 s	1.14 s	1.10 s
		NC	1.19 s	1.12 s	1.14 s	1.15 s

**Figure 5:**
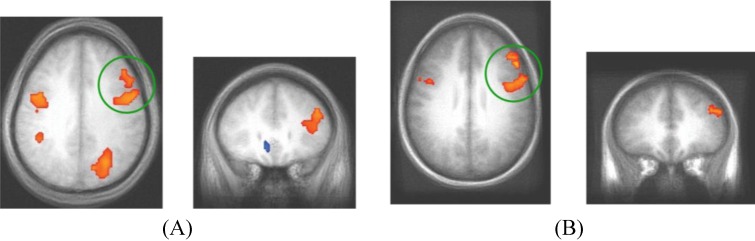
fMRI contrast of C *vs*. NC trial types. (**A**) For presentation, Dataset 1 was thresholded at *t* > 3.5 with a minimum cluster size of 300mm^3^. The peak coordinates of activity are (39, 21, 18) and (43, 1, 33); (**B**) For presentation, Dataset 2 was thresholded at *t* > 3 with a minimum cluster size of 800mm^3^ (DLPFC regions in both datasets are evident at more stringent *t* > 4.0 thresholds, the lower thresholds were used to show consistency across the replication). The peak coordinates of activity are (48, 3, 33) and (46, 33, 27). The consistent regions of activity across studies occur in the right DLPFC which we hypothesize corresponds to the operation of the Decision Module on these trials.

Neural activity differences between the C and NC trials were assessed in both experiments and shown in Figure 5. Given that the Decision Module is hypothesized to be active on every trial, for each dataset, we restricted the analysis to a functionally defined ROI based on all cross-subject trial-evoked activity. This smaller volume allowed for greater sensitivity than is afforded when searching the entire brain for trial-evoked activity. The grouped functional ROI was used to mask each individual subject’s contrast of competition-related activation. The resulting t-test then used the masked functional dataset to isolate significant clusters of activity (*t* > 3.5, cluster > 300 mm^3^). The regions shown reflect brain regions where C trials evoked more neural activity than NC trials. While there were regions of reliable differential activity across experiments (Table 4), the right DLPFC and bilateral motor cortex was found to exhibit greater activity for C trials in both datasets. These brain regions reflect candidate areas for the neural basis of the Decision Module where competition between the two category systems is resolved and the consistent activity observed in DLPFC across datasets indicates this are is likely of particular importance to this process. 

**Table 4:** Cluster information for the fMRI contrast of C *vs*. NC trial types (Figure 5) in Dataset 1 and Dataset 2.

Anatomical label	*x*	*y*	*z*	cluster size (mm^3^)	max *T*-value
**Dataset 1**					
Right Lingual Gyrus (BA19)	−27.1	75.5	−8.9	17,141	27.3
Left Middle Occipital Gyrus (BA19)	33.7	78.2	−8.8	16,328	26.0
Right Precuneus (BA7)	−24.2	63.5	39.8	7625	19.9
Right Middle Frontal gyrus (BA13)	−38.7	−21	18.4	6484	17.2
Right Inferior Frontal Gyrus (BA6)	−42.8	1.4	32.7	4656	15.4
Left Putamen	21.4	−1.1	10.4	4141	18.5
Left Inferior Parietal Lobule (BA7)	33.2	56.3	40.7	3875	18.0
Left Precentral Gyrus (BA6)	41.3	4.6	34	2422	15.0
Left Caudate	−11.3	−1.4	10.5	2266	20.71
Right Medial Frontal Gyrus (BA6)	−0.9	−9.7	45.7	2094	18.8
Left Cingulate Gyrus (BA23)	0.4	32.3	26	1906	16.6
Right Thalamus	−7.2	17.8	14.2	1000	19.5
Left Anterior Cingulate (BA24)	9	−28.9	−2.8	953	−14.5
Right Precentral Gyrus (BA6)	−32.4	10.9	56.7	703	20.3
Right Thalamus	−6.6	20.9	−3.6	531	9.50
Left Middle Temporal Gyrus (BA39)	47.2	78.7	25.8	531	−16.7
Left Insula (BA13)	34.3	9.1	15.6	500	10.5
Right Anterior Cingulate (BA32)	−1.1	−40.3	−1.8	312	−14.3
Left Declive	9.7	74.5	−20.3	312	11.7
					
**Dataset 2**					
Left Lingual Gyrus (BA19)	29	73	−9.1	21,797	29.0
Right Lingual Gyrus (BA19)	−33.4	76.4	−5.6	15,016	32.0
Left Postcentral Gyrus (BA3)	41.9	27.2	53.1	10,672	24.1
Right Inferior Frontal Gyrus (BA6)	−48.4	−2.8	33.2	3641	18.8
Right Middle Frontal Gyrus (BA6)	−40.7	−0.6	55.9	1141	20.2
Left Middle Frontal Gyrus (BA9)	48.8	−5.8	35.3	1031	17.1
Right Middle Frontal Gyrus (BA9)	−46.4	−33.2	27.2	984	21.8
Left Superior Frontal Gyrus (BA8)	19.9	−23.3	43.4	844	−11.6

To accomplish the competition resolution process, the role of the DLPFC on these trials may be to actively inhibit one system so that the other system can send activation to the motor system to make the appropriate motor plan. This type of inhibitory role of the DLPFC has been previously observed using fMRI with task-switching paradigms where one task-response needs to be inhibited to allow the other to progress [31]. Damage to prefrontal cortex has been reported to lead to impaired category learning, particularly due to difficulty identifying the optimal strategy [32]. Handling predictions of multiple category learning systems requires a process like this to be involved in managing the multiple sources of information. However, in order to identify neural activity associated with this process it is necessary to construct a model such as PINNACLE that embeds specific hypotheses about the mental state of each participant on each trial so that trials differentially dependent on this process can be contrasted.

### 4.5. Result 3: Exploring Off-System Activity

On each trial, PINNACLE identifies either the RB or II system as winning the competition and driving the behavioral response. As noted in Section 3, this raises an interesting question about how feedback is handled in the model and specifically whether the “off system” (the category learning system that is currently not driving the response) learns from trial feedback. We first investigated this question by comparing the fit of different versions of PINNACLE that used different feedback mechanisms. It was found that feedback returning to both systems on every trial to allow for updates and improved future category judgments provided the best fit to behavioral data. The availability of fMRI data during category learning provided another method of further exploring this issue.

Trials in Dataset 1 and 2 were defined based on whether the off-system correctly predicted category membership. As discussed in Section 3, the experimental feedback is tied to the subject’s response, but under the current learning mechanism it is possible for the non-dominant system to acquire category knowledge covertly. For example, if an “A” stimulus is predicted to be an “A” both by the highly confident RB system and a less confident II system, the RB system of this subject would have received feedback that the response was correct. Because the II system also receives feedback, it would also update its representation by decreasing the noise around the category bound accordingly. In this manner, it is possible for the non-dominant, off-system to effectively learn the categorical boundary despite the output response being attributed to the dominant system. This process allows for more rapid strategy switching when the suboptimal strategy has come to control the decision processes. If a participant is using an RB strategy for an II task, the II system is still learning so that when the RB system eventually makes incorrect predictions, the II system can start winning the competition rapidly.

**Figure 6:**
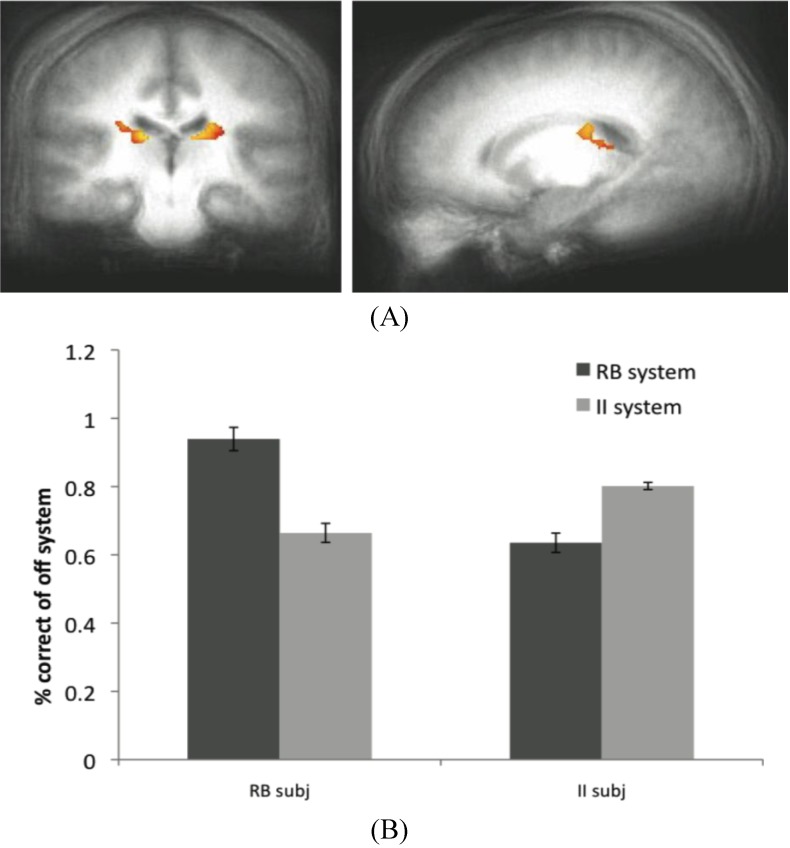
(**A**) Off system activity in RB subjects. In the RB participant group in Experiment 2, trials were marked as correct or incorrect according to the predictions of the II system when PINNACLE predicted the RB system was active. Activity within the caudate ROI is significantly active in RB participants during II trials when the II system was not selected. These posterior caudate regions are consistent with the hypothesized “II network”, so the observation of activity here suggests that the II system is operating simultaneously with the RB system in these participants; (**B**) Accuracy of the off-system in RB and II subjects. The correct and incorrect designations are based on the off-system’s predictions, not the overt feedbackto given the subject.

By analyzing activity in brain regions associated with the off system, we hypothesized that it might be possible to identify neural activity associated with this otherwise covert process (off system learning). In Dataset 1, there was no evidence of off-system activity either in the MTL or the caudate for II or RB subjects, respectively. However, in Dataset 2, perhaps due to the higher variability in the category stimulus space, there was evidence of off-system II activity in the caudate ROI for RB subjects (Figure 6A) based on contrasting successful and unsuccessful category predictions of the II module. Note that there is no external measure of II predictions other than our estimates provided by fitting PINNACLE to the behavioral data. The accuracy of the off-system responses for Dataset 2 is shown in Figure 6B. The off-system was more accurate when it was associated with the appropriate participant group. That is, in RB subjects when the RB system was the off-system, those trials were more accurate than when the II system was the off-system. The converse was true for the II subjects. This pattern suggests that learning in the off-system leads to better performance when the off-system is the eventual optimal system. 

The data from the RB subjects in Dataset 2 supports the idea that the II system, while not overtly responsible for behavior, is not only active in these subjects, but appears to be utilizing the feedback to update its category representation. The posterior regions of the body of the caudate that were more active for correct than incorrect off-system trials in these subjects are identical to the regions typically active during successful on-system II category learning. Concurrent with the success of the RB system in these subjects (and corresponding MTL activity), the II system appears to be operating in parallel through activity in the posterior caudate body.

While a similar result was not observed in the MTL when the RB system was the off-system in II subjects, this does not rule out the notion that both systems are capable of learning simultaneously. However, because off system activity was only observed in one condition in one dataset, this finding should be considered preliminary evidence for this phenomenon. The absence of a more consistent finding may reflect a weak signal or some variability in trial type sorting, possibly due to the fact that the PINNACLE instantiation of the RB model does not allow for as wide a range of hypotheses about the category boundary as the participants may have considered. This type of analysis is potentially promising as an analytic technique because it provides another way to see a connection from internal states of the computational model and evoked neural activity associated with otherwise covert cognitive processes. For this approach to work, the model has to provide a highly accurate method of sorting neural events and increasing the specificity of the model would likely enhance the approach. The caudate activity observed in the off-system provides intriguing evidence that PINNACLE’s mechanism of feedback returning to both systems has some neural reality consistent with the competitive model fitting described in Section 3.6. 

## 5. General Discussion

Categorization can be defined broadly as the act of responding differently to objects and events in the environment based on their belonging to separate classes or groups. Objects within a category typically share certain behavioral characteristics or physical features. Category learning refers to the cognitive processes that extract similar features from a collection of stimuli to extract the category structure and allow for accurate categorization of future novel stimuli. This ability reduces the need for a separate response to each object in the world, making behavior more economical. Given the important role of categorization in everyday life, it is not surprising that there is a long history of study on the underlying cognitive operations that mediate this skill. Because the efficiency gained by grouping stimuli into categories to infer likely properties and responses, it is also not surprising that the brain contains more than one mechanism for extracting category structure.

Evidence for multiple category learning systems comes from experimental work, neuropsychological studies and more recently from functional neuroimaging studies using fMRI. Each of these approaches suggests there is an important difference between category learning depending on the MTL *versus* depending solely on cortico-striatal circuits. However, having multiple systems within the brain contributing to a learning process complicates the process of investigating human category learning. While experimental tasks can encourage one strategy based on one system, it is clear that participants will try multiple strategies and switch strategies during the learning process ([9,10,18,19,33]), as we have demonstrated previously on a block-by-block basis [18] and now with PINNACLE’s trial-by-trial predictions.

Using model-based predictions to organize neuroimaging data improves on existing analysis techniques by making to possible to isolate activity associated with strategy-specific behavior. Rather than grouping activity based on the imposed category structure, this kind of mathematical characterization of strategy-use can identify participants who are using a sub-optimal strategy or assess how well the appropriate strategy is being utilized. This can be done potentially on a trial-by-trial basis to enhance the analysis of physiological data such as fMRI. We identified a number of regions using this approach that had not been observed in the success-based fMRI analysis used previously. Specifically, PFC and visual association cortex activity was associated with trials that mostly clearly reflected the use of RB and II strategies, respectively, based on fit estimates. The PFC regions are thought to function in conjunction with the MTL in RB learning. The visual association cortex is connected to the posterior body of the caudate and thought to support II learning. The roles of these areas in the two category learning systems were first anticipated in the description of COVIS [6]. However, the visualization of the additional regions would not have been possible without the application of the PINNACLE model (which is based on the COVIS theory) to guide fMRI data analysis.

The modeling framework of PINNACLE implements an architecture to incorporate two active category learning systems in the healthy brain. Both systems are implemented as simple DBT models as approximations to the functions of these types of learning. A richer model of human category learning could implement computational approaches to RB and II that aim to capture more of the processing within these systems: hypothesis testing for RB and a more flexible perceptual space labeling system for II. For the analysis here, the simpler models capture the category structures that were administered experimentally and the analysis serves to both identify neural components of human category learning and also to demonstrate the potential of combined computational modeling and fMRI techniques.

A key element of PINNACLE is to propose a specific computational architecture for integrating the two competing category learning component systems. We proposed a convergence area termed a Decision Module where competition between the two systems would be resolved and an output response selected. This is a necessary component to a multi-system model that will tend to be overlooked in approaches primarily aimed to dissociate the two types of category learning. By using the PINNACLE model to identify subsets of trials that should require more involvement of this module, we were able to provide a possible neural correlate of this process and simultaneously provide some corroborating evidence for it. The dorsal PFC is a highly plausible neural basis of the Decision Module because of its general role in cognitive control and a region that can flexibly select, maintain, update or transform information in the service of a goal [34,35]. The need for a method for resolving competition also raises a related question of how to handle trial feedback in a competitive multiple-model system. Simulation analysis to fit human behavior indicated that when feedback is available to both systems, the model most closely matches performance. This idea was further explored in the fMRI data and preliminary results suggested that in some cases, it is possible to see neural correlates of activity indicative of learning in the category learning system not driving the most recent behavioral response.

The application of model-based fMRI analyses has great potential for testing hypotheses about the mechanistic underpinnings of the multiple category learning systems that co-exist in the brain. In its current state, PINNACLE enabled explorations into RB and II trial-by-trial system engagement, system competition resolution, and feedback incorporation. As discussed above, future direction of this type of research could include mechanistic improvements to the RB and II systems that better reflect the neurobiological properties of the MTL and cortico-striatal systems (see [36]). The question of covert learning in the off-system may also merit further investigation. We found models with off-system learning to provide the best fit to human behavior and found some evidence for learning in the II system even when performing an RB task (with an RB strategy). However, there remain complications with handling off-system feedback. For example, it is unclear how to handle a case such as when the RB system predicts “A” while the II system predicts “B” but the RB system drives the response (“A”), which then turns out to be incorrect. In the current implementation of PINNACLE, the II system should increase its future confidence, but the global feedback signal was negative (during the task the word “Wrong” appears). To incorporate positive feedback would require reinterpretation mechanisms which we did not postulate as part of PINNACLE here (in the absence of a neural basis for this process). In addition to improving the category representations of the RB and II systems, more detailed feedback handling mechanisms may also be necessary to realize a computational model with strong reflection in the brain’s neurophysiology.

## 6. Conclusions

Together, the combination of neuroimaging and modeling data presented here demonstrate the utility of these integrated approaches for revealing the inner operations of the human brain in a way that neither alone could achieve. This symbiotic relationship between model development and fMRI data analysis advances both our understanding of RB and II category learning systems in the brain, and demonstrates the great potential this approach has for testing hypotheses about cognitive processes outside our awareness. 

## References

[1] Ashby F.G., Maddox W.T. (1990). Integrating information from separable psychological dimensions. J. Exp. Psychol. Hum. Percept. Perform..

[2] Ashby F.G., Maddox W.T. (1992). Complex decision rules in categorization: Contrasting novice and experienced performance. J. Exp. Psychol. Hum. Percept. Perform..

[3] Ashby F.G., Maddox W.T. (1993). Relations between prototype, exemplar, and decision bound models of categorization. J. Math. Psychol..

[4] Maddox W.T., Ashby F.G. (2004). Dissociating explicit and procedural-learning based systems of perceptual category learning. Behav. Process..

[5] Maddox W.T., Ing A.D. (2005). Delayed feedback disrupts the procedural-learning system but not the hypothesis-testing system in perceptual category learning. J. Exp. Psychol. Learn. Mem. Cogn..

[6] Ashby F.G., Alfonso-Reese L.A., Turken A.U., Waldron E.M. (1998). A neuropsychological theory of multiple systems in category learning. Psychol. Rev..

[7] Ashby F.G., Valentin V.V., Lefebvre H.C.C. (2005). Multiple Systems of Perceptual Category Learning: Theory and Cognitive Tests. Categorization in Cognitive Science.

[8] Zeithamova D., Maddox W.T. (2006). Dual-task interference in perceptual category learning. Mem. Cogn..

[9] Maddox W.T., Ashby F.G., Ing A.D., Pickering A.D. (2004). Disrupting feedback processing interferes with rule-based but not information-integration category learning. Mem. Cogn..

[10] Maddox W.T., Filoteo J.V. (2001). Striatal contributions to category learning: quantitative modeling of simple linear and complex nonlinear rule learning in patients with Parkinson’s disease. J. Int. Neuropsychol. Soc..

[11] Nomura E.M., Maddox W.T., Filoteo J.V., Ing A.D., Gitelman D.R., Parrish T.B., Mesulam M.M., Reber P.J. (2007). Neural correlates of rule-based and information-integration visual category learning. Cereb. Cortex.

[12] Poldrack R.A., Clark J., Pare-Blagoev E.J., Shohamy D., Creso Moyano J., Myers C., Gluck M.A. (2001). Interactive memory systems in the human brain. Nature.

[13] Seger C.A., Cincotta C.M. (2006). Dynamics of frontal, striatal, and hippocampal systems during rule learning. Cereb. Cortex.

[14] Ashby F.G., Gott R.E. (1988). Decision rules in the perception and categorization of multidimensional stimuli. J. Exp. Psychol. Learn. Mem. Cogn..

[15] Maddox W.T., Ashby F.G., Waldron E.M. (2002). Multiple attention systems in perceptual categorization. Mem. Cogn..

[16] Ashby F.G. (1992). Multidimensional Models of Perception and Cognition.

[17] Ashby F.G., Waldron E.M. (1999). On the nature of implicit categorization. Psychon. Bull. Rev..

[18] Nomura E.M., Maddox W.T., Reber P.J., McNamara D.S., Trafton J.G. (2007). Mathematical models of visual category learning enhance fMRI data analysis. Proceedings of the 29th Annual Conference of the Cognitive Science Society.

[19] Maddox W.T., Ashby F.G., Bohil C.J. (2003). Delayed feedback effects on rule-based and information-integration category learning. J. Exp. Psychol. Learn. Mem. Cogn..

[20] Estes W.K., Straughan J.H. (1954). Analysis of a verbal conditional situation in terms of statistical learning theory. J. Exp. Psychol..

[21] Press W., Teukolsky S., Vetterling W., Flannery B. (1992). Numerical Recipes in C.

[22] Schultz W. (1992). Activity of dopamine neurons in the behaving primate. Semin. Neurosci..

[23] Wickens J. (1990). Striatal dopamine in motor activation and reward-mediated learning: Steps towards a unifying model. J. Neural Transm. Gen. Sect..

[24] Maddox W.T., Love B.C., Glass B.D., Filoteo J.V. (2008). When more is less: Feedback effects in perceptual category learning. Cognition.

[25] Ashby F.G., O’Brien J.B. (2007). The effects of positive *versus* negative feedback on information-integration category learning. Percept. Psychophys..

[26] Cox R.W. (1996). AFNI: Software for analysis and visualization of functional magnetic resonance neuroimages. Comput. Biomed. Res..

[27] Collins D.L., Neelin P., Peters T.M., Evans A.C. (1994). Automatic 3D intersubject registration of MR volumetric data in standardized Talairach space. J. Comput. Assist. Tomogr..

[28] Insausti R., Juottonen K., Soininen H., Insausti A.M., Partanen K., Vainio P., Laakso M.P., Pitkanen A. (1998). MR volumetric analysis of the human entorhinal, perirhinal, and temporopolar cortices. AJNR Am. J. Neuroradiol..

[29] Reber P.J., Wong E.C., Buxton R.B. (2002). Encoding activity in the medial temporal lobe examined with anatomically constrained fMRI analysis. Hippocampus.

[30] Stark C.E., Okado Y. (2003). Making memories without trying: Medial temporal lobe activity associated with incidental memory formation during recognition. J. Neurosci..

[31] Sylvester C.Y., Wager T.D., Lacey S.C., Hernandez L., Nichols T.E., Smith E.E., Jonides J. (2003). Switching attention and resolving interference: fMRI measures of executive functions. Neuropsychologia.

[32] Schnyer D.M., Maddox W.T., Ell S., Davis S., Pacheco J., Verfaellie M. (2009). Prefrontal contributions to rule-based and information-integration category learning. Neuropsychologia.

[33] Ashby F.G., Crossley M.J. (2010). Interactions between declarative and procedural-learning categorization systems. Neurobiol. Learn. Mem..

[34] Duncan J. (2001). An adaptive coding model of neural function in prefrontal cortex. Nat. Rev. Neurosci..

[35] Miller E.K., Cohen J.D. (2001). An integrative theory of prefrontal cortex function. Annu. Rev. Neurosci..

[36] Ashby F.G., Maddox W.T. (2011). Human category learning 2.0. Ann. N. Y. Acad. Sci..

